# Sensitive Metal Oxide-Clay Nanocomposite Colorimetric Sensor Development for Aflatoxin Detection in Foods: Corn and Almond

**DOI:** 10.1021/acsomega.1c00750

**Published:** 2021-06-03

**Authors:** Nishtha Khansili, Prayaga Murali Krishna

**Affiliations:** Department of Basic and Applied Science, National Institute of Food Technology Entrepreneurship and Management (NIFTEM), Haryana 131028, India

## Abstract

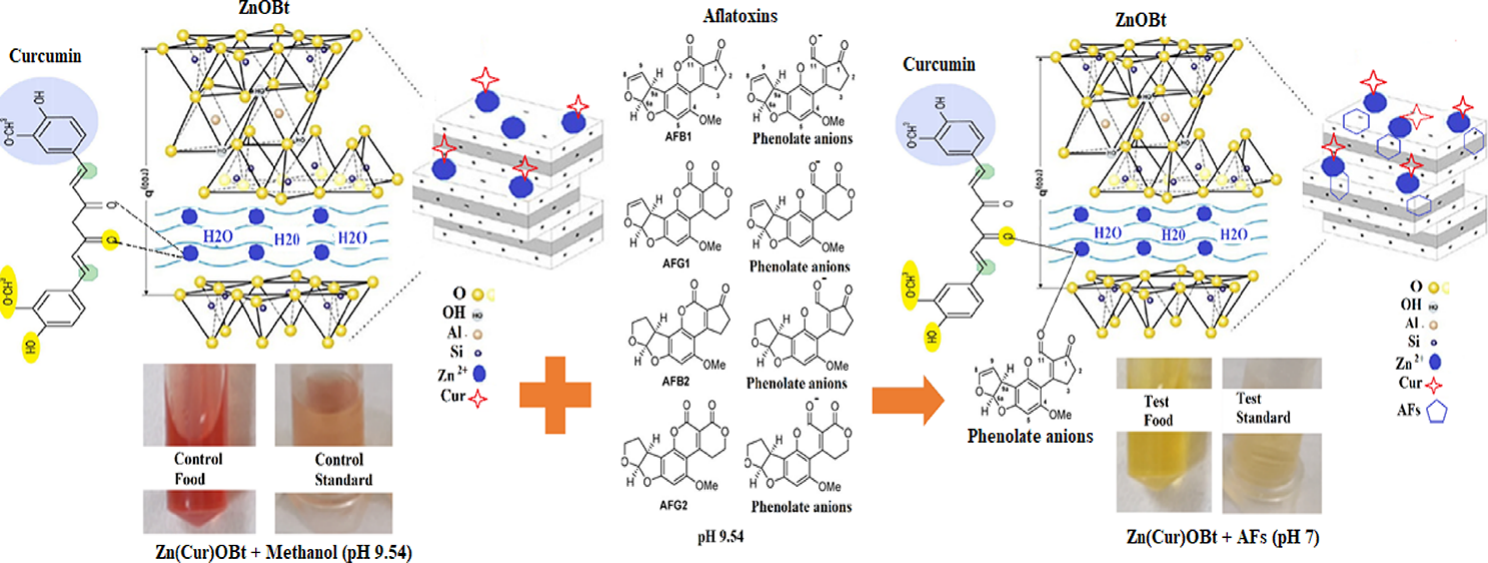

The work reports on zinc oxide bentonite nanocomposite (ZnOBt) chemical route synthesis, characterization, and investigation of curcumin (Cur) functionalization for a label-free colorimetric detection of total aflatoxins (AFs) in foods. XRD of ZnO nanoparticles (NPs) confirmed the wurtzite structure (2θ = 36.2°) and that of ZnOBt showed the intercalated interlayer composite phase. The Debye–Scherrer relation calculated the crystallite size as 20 nm (ZnO) and 24.4 nm (ZnOBt). Surface morphology by SEM exhibited flower-like hexagonal, rod-shaped ZnO NPs on the bentonite surface. Colorimetric reaction involved two-stage redox reactions between ZnOBt and dye Cur followed by AFs phenolic group and Zn(Cur)OBt. Cur gets oxidized at its diketone moiety in the presence of ZnOBt to form a red colored complex Zn(Cur)OBt, which further scavenge protons from AFs phenolic group, and gets oxidized to AFs-Zn(Cur)OBt (yellow). Binding of AFs-Zn(Cur)OBt is characterized by FT-IR ascribed to C–H bending (1966.615 cm^–1^), O–H stretching (3256.974 cm^–1^), and C=O stretching (1647.362 cm^–1^). ^1^H NMR chemical shifts (δ) (ppm) showed an increase in proton at the aliphatic region (0 to 4.4) while removal of proton in ether at 4.4 to 6 regions. Job plot calculation using UV–Vis data resulted in a higher total AF binding coefficient of Zn(Cur)OBt (*K*_a_ = 3.77 × 10^6^ mol^–1^ L) compared to Zn(Cur)O (*K*_a_ = 0.645 × 10^6^ mol^–1^ L) as well as a molar ratio of 1:1 by the Benesi–Hildebrand plot equation. Corn and almond food samples showed the total AFs LOD of 2.74 and 4.34 ppb, respectively. The results are validated with standard LC/MS-MS in compliance with MRL value as per the regulatory standard (EU).The NP-based method is facile and rapid and hence can be utilized for onsite detection of total AFs in foods.

## 1 Introduction

Aflatoxins have major subtypes as B1, G1, B2, and G2 and are carcinogenic, heterocyclic, oxygenated, stable difuranocoumarins metabolites of *Aspergillus flavus* and *Aspergillus parasiticus*, prevalent mostly in cereals, nuts, and tree nuts.^1^ Lately, 20 different subtypes of AFs are known, which occur naturally in feed and foods. Whilst 13 of them are naturally produced by mold, the rest are known as toxic metabolized derivatives produced by animals, microorganisms, and humans.^2^ Out of them, aflatoxin M1 (AFM1) is very important and requires major attention to combat its prevalence in dairy products. Meanwhile, other derivatives are also prevalent since they can rapidly invert to the potent AFB1 and can become intermediate for synthesis of other potent mycotoxins. Hence, due to the higher risk evidence of parent AFs and its derivative molecule as a carcinogen for animals and humans, the International Agency for Research on Cancer (IARC) reported AFs as a class I carcinogen.^3^ The analysis by the Council for Agriculture Science and Technology (CAST) reported that, each year, a significant proportion of world’s crop (especially corn) and oilseed supply would be contaminated with AFs.^4^ As a result of which, several countries have adapted distinct maximal residual action limit for AFs for food in the range of 8 to 30 ppb.^5−8^

Of late there are different chromatographic and mass spectroscopic analytical techniques available for AFs detection in agricultural food crops and feeds.^5,9,10^ Additionally, commercial rapid detection kits^11−17^ are effortless and quick. They involve high-grade AFs-selective antibody, aptamer, and DNA-enzyme-based conjugates for attaining higher selective and sensitive detection. However, due to their instability and tedious production, cost and attainment of these conjugates have always been a menace. A label-free biosensor holds the solution to this problem by offering a simple target mediated approach for cost-effective, time-based, and facile detection. Further, for AFs detection, generally two or more AFs simultaneously contaminate the food grain,^11^ and many immunoassays are specific to detect individual aflatoxins^14−17^ and hence cannot quantify AFs together in foods. Literature review indicated a few label-free analytical reports^18^ using spectroscopic techniques for AFs detection, like Raman spectroscopy,^19^ hyperspectral system,^20^ and near-infrared spectroscopy, indicating good results in foods. However, simple UV–Vis, which is a widely available instrument, is reported less for AFs screening.

Lately, there are a few colorimetric method-based reports for quantification of macro organic molecules like pollutants and^21^ heavy metals^22,23^ by colorimetric binding with supported metal oxide NPs on clay composites. The detection involved estimating the direct output signal proportional to the interaction caused between the analyte and supported composite material. Among other metal oxide nanomaterials, ZnO has several advantages due its wide band gap (3.35 eV), low cost, high aspect ratio, and improved optoelectronic properties. It shows unique structural properties like it has a second coordination sphere, which entails itself in hydrogen binding likely with ligands, water molecules, and hydrophobic cores, for improved binding results.^24^ Additionally, it was reported that catalytic activity of these oxide materials can be enhanced when they are linked or immobilized on a natural clay material such as bentonite, which has a higher surface area.^25^ Moreover, introducing nanoparticles with other substrates/fillers would result in composites with novel and enhanced properties that cannot be achieved by the individual components. Clay minerals are excellent fillers for metal composites. Clay minerals have been gaining attention of manufacturers and scientists throughout the world because of their low cost, high specific surface area, chemical/mechanical stability, them having a variety of surfaces, higher adsorption, and structural properties.^26^ Gumpu et al.^27^ reported increased specific reaction and electron transfer rate between the target analyte and metallic nanoparticle-based clay nanocomposite on the electrode surface in aqueous solution.^23,28,29^ Treatment of bentonite with ZnO NPs are in accordance with the study published by Rashid et al.,^25^ which reports the binding efficiency of bentonite clay increased with ZnO NPs for AFs within biological cells. It is essentially due to Zn ions on the bentonite surface, which creates a AFs-Zn chelation complex at the dicarbonyl functional group of AFs.^30,31^

**Scheme 1:**
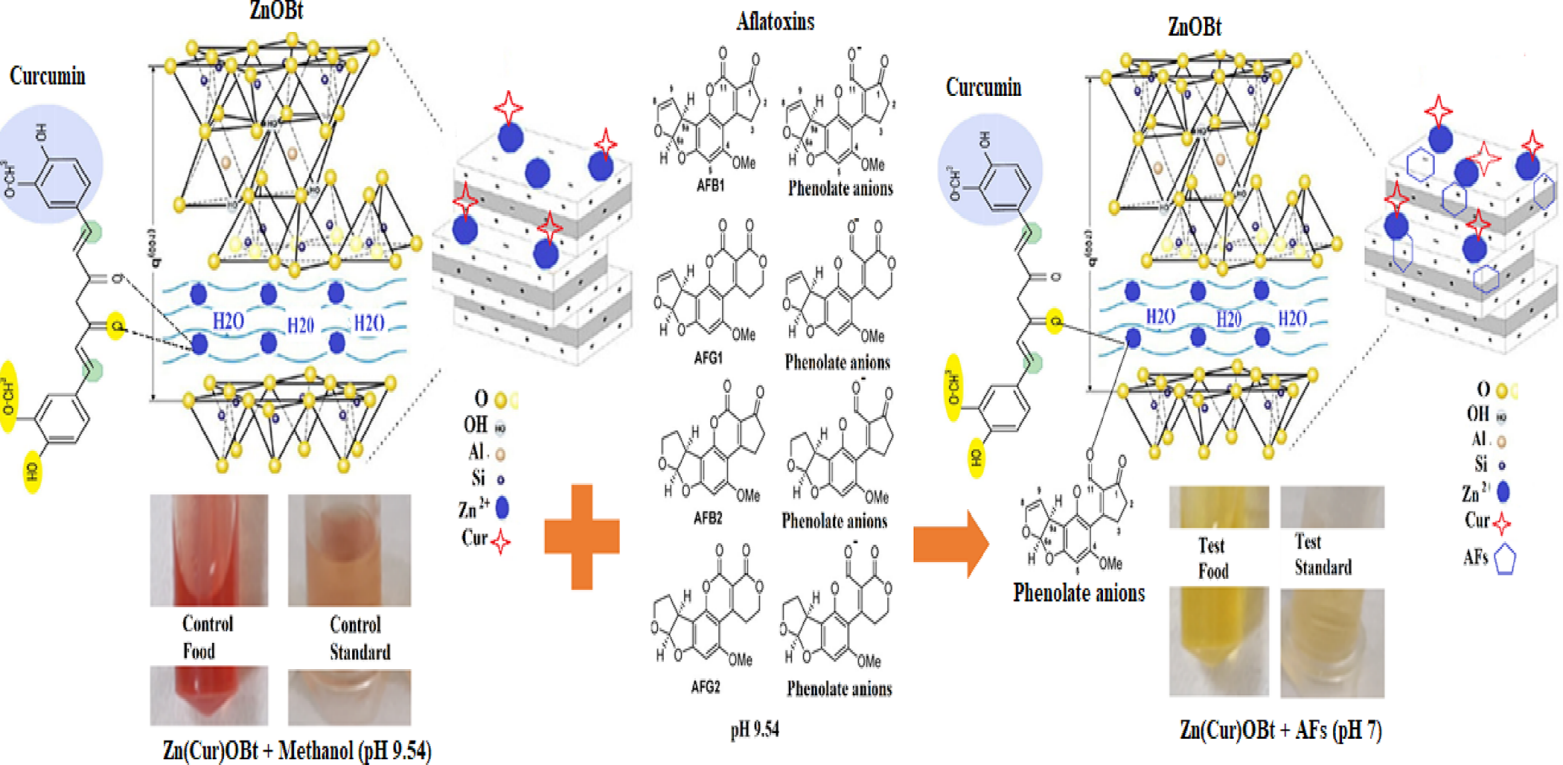
Schematic Representation for Colorimetric Detection of Total AFs by the Zn(Cur)OBt Composite at Alkaline pH (9.54) Zn(Cur)OBt in the absence of AFs remained red in control samples and changes to yellow color and forms AFs- Zn(Cur)OBt, reducing alkaline pH to neutral pH in the presence of AFs.

However, ZnOBt sensitization toward visible light for detecting AFs requires splitting of band gap into several sub-gaps and can be performed by incorporation of fullerenes,^46^ organic materials,^45^ and polymers.^47−49^ The Cur dye-modified ZnO-based nanocomposite studies for optical, colorimetric, and electro-chemical sensors are not explored much. In this work, we attempted to prepare a new Cur-functionalized ZnOBt nanomaterials, which is termed here as Zn(Cur)OBt, and apply it for developing a label-free visible sensing scheme for AFs as depicted in Scheme 1 to achieve simple, low cost, easier, rapid, sensitive and selective results. The curcuminoid complex is widely acknowledged for its medicinal role in therapy and pharmacy and for major diseases.^32−34^ It has very unique photophysical and fluorescence characteristics.^35^ As a fluorescent probe, it has been widely realized to be installed in several sensing schemes^36−38^ and acts as a reducing agent with metal NPs^39^ and nanorods.^40^ It forms a chelating complex with different metal ions^41−43^ and zinc salt by forming a Zn-Cur complex.^44^ Thus, functionalizing ZnOBt with Cur may enhance the absorption and luminescence properties of ZnO and overall adsorption efficiency of ZnOBt and hence can be utilized for colorimetric total AFs detection in our colorimetric sensor studies.

## 2 Results and Discussions

### 2.1 Characterization of the ZnOBt Nanocomposite

**Figure 1:**
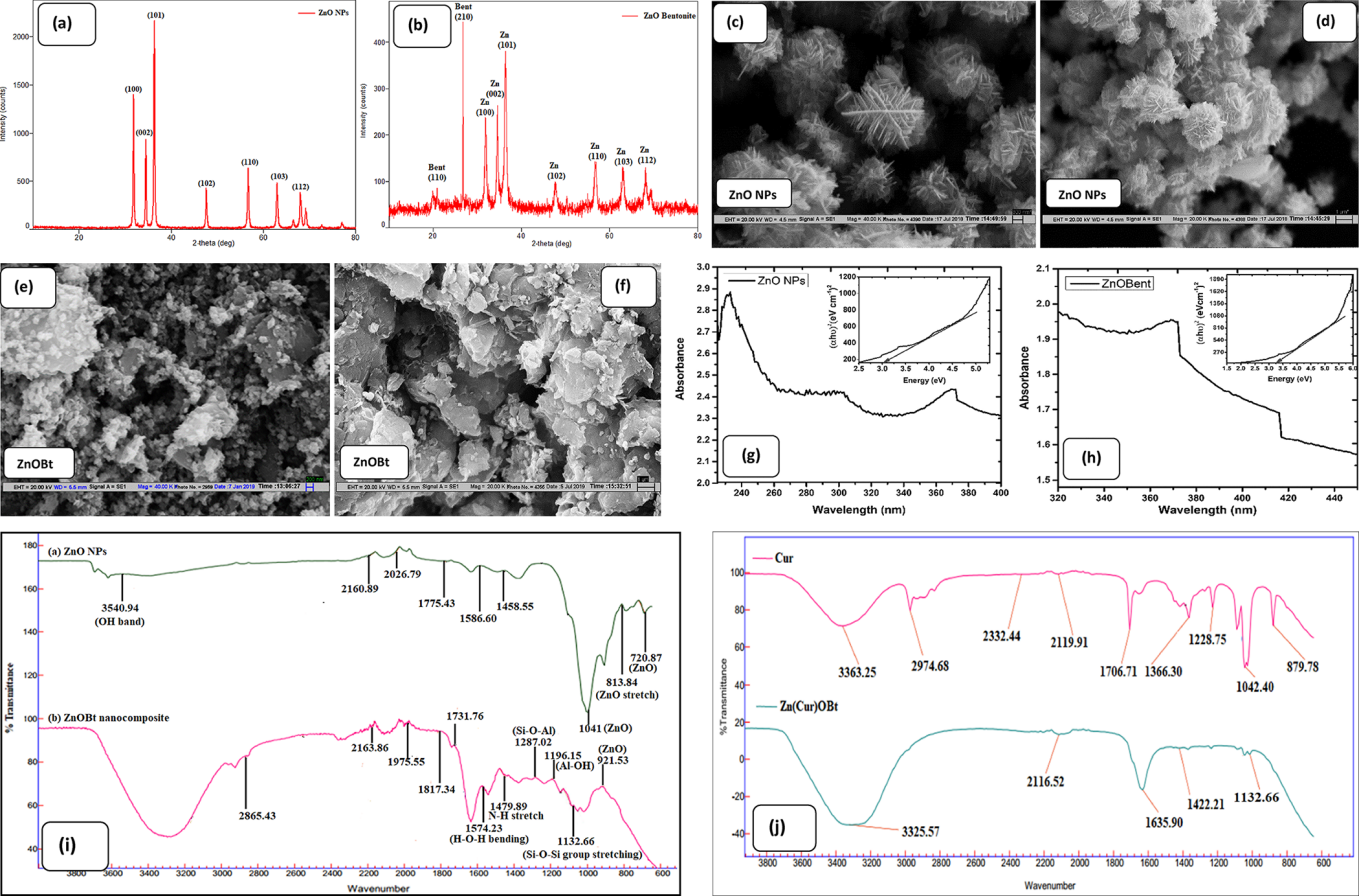
XRD of (a) ZnO and (b) ZnOBt. SEM images of (c, d) ZnO NPs and (e, f) ZnOBt. UV–Vis spectra (inset, Tauc plot) of (g) ZnO NPs and (h) ZnOBt. FT-IR spectra of (i) ZnO NPs and ZnOBt and (j) Cur and Zn(Cur)OBt.

The crystallite phase, surface morphological, colloidal stability, optical properties and functional groups of ZnOBt required for AFs colorimetric assay were characterized by XRD, SEM, UV–Vis, and FT-IR. XRD confirmed the crystallite wurtzite phase of nanosized ZnO NPs and the hexagonal phase of bentonite in the ZnOBt nanocomposite. The crystallite sizes of ZnO NPs and ZnOBt were calculated by X-ray beam interference using Bragg’s law or the Debye–Scherrer equation (*D* = 0.9λ/β cos θ) as 20 and 24.4 nm, respectively. Figure 1a,b shows the XRD spectra of ZnO and the ZnOBt nanocomposite. The maximum intensity peak (2θ) at 36.2° and 26.5° indexed to crystalline planes at Miller indices of (101) for ZnO and (210) for bentonite was observed in the ZnOBt nanocomposite. The values were matched and found in accordance with the standard (JCPDS file number 750526).^50−52^ Further, XRD of ZnOBt revealed broadening of peaks as compared with bare ZnO NPs. This was observed due to potency of ZnO NPs to bridge the adjacent silicate units present in interlayers, similar to a behavior shown by different oxides in the intermittent layers of bentonite as conferred by Sonawane et al.^53^ Additionally, in comparison with the natural clay, ZnOBt nanoclay intercalation is more advanced in a higher 2θ region (30–40°), showing generation of the intercalated composite.^54^

SEM morphologies obtained similar results indicative of intercalation for bare ZnO NPs on the surface and interlayers of bentonite. ZnO NPs showed flower-like, spherical, hexagonal rods (Figure 1c,d) as shown in our previous study,^55^ while the ZnOBt nanoclay exhibited a platelet structure of bentonite in tactoid form with uniform stacking of flower-shaped ZnO nanostructures on the surface and interlayers as shown in Figure 1e,f. Embedded ZnO NPs in the interlayers of bentonite for the ZnOBt nanocomposite also indicated a decrease in size, conferred with XRD and surface area morphology of ZnO NPs as per unit surface area of bentonite clay mineral.^56^ This was further evidenced with UV–vis spectra, where a blue shift of 5 nm was observed for bare ZnO NPs at 373–368 nm for the ZnOBt nanocomposite. As shown in Figure 1g,h, the band gap was drawn using Tauc plot and the *E*_g_ value was found to be higher for ZnOBt (3.35 eV) than bare ZnO NPs (3.05 eV). The results were indicative of a decrease in particle size for ZnO NPs supported within the clay matrix due to electron confinement at the nanoscale, with so-called “quantum size effect”, as compared with unsupported bare ZnO NPs.^56^ The discrete and uniform distribution of in situ prepared ZnO NPs, anchored on the bentonite surface, was further confirmed by chemical and functional bonds using FT-IR analysis. It further affirmed the improved optical property and advanced surface-active sites of ZnOBt.

As shown in Figure 1i, the FT-IR spectra of ZnO NPs and ZnOBt were compared, indicating the presence of bentonite and ZnO NPs in the intercalated nanocomposite. The composite showed the band location at around 1500–1350 cm^–1^ for N–H stretch of bentonite and at around 800–400 cm^–1^ for ZnO stretch.^57,58^ The absorption in the region around 1574 cm^–1^ and band centered at 1132 cm^–1^ was related to the H–O–H bending vibration of water and siloxane (−Si–O–Si−) group stretching for bentonite.^31,51,59^ The capping agent and stabilization of as-synthesized ZnO NPs may be due to the coordination of ZnO NPs with C=O and −OH groups.^49,60^ Other bands in the region between 1200 and 800 cm^–1^ were identified as Si–O–Al, Si–O–Si, and Al–OH–Mg^31^ peaks at 921.53, 1132.66, and 1574.23 in the ZnOBt nanocomposite. Thus, characterization revealed ZnOBt synthesized for prevented free release of supported Zn NPs into the environment, prevented agglomeration, and effective colorimetric AFs detection in the food matrix in this study. Further, to functionalize ZnOBt with Cur, fingerprint vibrations were studied for both Cur and Zn(Cur)OBt, as shown in Figure 1j, to support their interaction. Table 1 shows the molecular band of the bare material and composite used for colorimetric detection. ZnO NPs showed no functional group at around 3500 cm^–1^ nor at 1600 cm^–1^. The absence of these bands indicates no O–H stretching vibration for the hydroxyl group and surface H–OH group bending vibration. Thus, no adsorption took place on the surface of bare ZnO NPs of any hydroxyl group.^64^ Meanwhile, the peak shift in ZnOBt and Zn(Cur)OBt from 720 and 813 cm^–1^ to 921 and 931 cm^–1^ was less dominant to indicate the altered Zn–O bond in the presence of bentonite and Cur.^65^ The n(OH) vibration at 3595 cm^–1^ in Cur indicated the frequency region of phenolic groups, shifted to a lower frequency band at 3363 cm^–1^ in Zn(Cur)OBt to indicate Zn(Cur)O binding. Cur has the prominent β-diketone group in its molecular structure, which create the metal-chelation ability of Cur.^42,66,67^ Khalil et al. reported the mass spectra of Zn-Cur showing a four-coordination complex formed at the β-di-keto system.^67^ This is due to the presence of a keto and enol tautomeric group in Cur. Thus, it indicated the formation of a charge transfer complex by forming a weak or strong bond with the Zn atom at the β-diketone moiety on the surface of ZnO. Figure 1j indicates the Cur spectra with no peak in the carbonyl region (1700–1650 cm^–1^) as reported earlier, neither in the solid nor in liquid state. It indicated that Cur exists mainly in the enol form; however, the peak at 1635 cm^–1^ for Zn(Cur)OBt was observed, which could be due to the formation of the n(C=O) group in Cur. Tayyari et al. stated that the broadness and intensity of the enol peak in Cur is dependent on the strength of the intramolecular hydrogen bond.^68^ It increases with a decrease in intensity and an increase in broadness of enol band location. Meanwhile, in Zn(Cur)OBt, there is a shift in ν (OH stretch) to the keto band at 3325 cm^–1^ and an increase in peak intensity. It indicated the formation of a chelate complex and the formation of Zn(Cur)OBt.

**Table 1:** Assignment of Important Infrared Spectral Bands (cm^–1^) for ZnO NPs, ZnOBt, Cur, and Zn(Cur)OBt in FT-IR

material	wavenumber (cm^–1^)	description for band described	reference
ZnO NPs	3540.94, 813.84, 720.87	sharp weak OH stretch, Zn–O stretch, Zn–O	(57, 58)
ZnOBt	3325.37, 1574.23, 1479.89, 1287.02, 1196.15, 1132.66, 921.53	H–O–H asymmetric and symmetric stretch, H–H bending, N–H stretch, Si–O–Al, Al–OH, SiO–Si, Zn–O	(31, 51, 53, 59, 60)
Cur	3363.25, 1706.71, 1366.30, 1228.75, 1042.40, 879.78	strong broad OH stretch, stretching vibration of alkene C=C and carbonyl C=O, C=C aromatic stretch, C–O phenolic band, C=O stretch, alkene C–H	(61)
Zn(Cur)OBt	3325.57, 2116.52, 1635.90, 1422.21, 1132.66	strong broad OH stretch, aromatic C–C vibrations bands of CO–Zn group, C=C aromatic stretch, SiO–Si	(62, 63)

### 2.2 AFs Sensor Mechanism and Binding Confirmation Characterization

The as-synthesized ZnOBt nanocomposite was further investigated for colorimetric application. It comprised functionalizing ZnOBt with Cur to detect not just AFB_1_ but also total standard AFs (AFB_1_, AFB_2_, AFG_1_, and AFG_2_ in the ratio of 1:0.1:0.3:0.03) in the food matrix. In order to create specific electrochemical bonds with Cur, the functional moiety of AFs is unable to cause direct discoloration of the dye due to the absence of ligand groups like nitro, sulfhydryl, or amino, as shown in the chemical structure of AFs in Figure 6d. Certain reports suggested that colorimetric methods^69^ based on discoloration of dye, adsorbed on the ZnOBt surface, causing electrochemical binding between the analyte (small molecule) and ZnOBt may serve as an alternative approach.^69^

Daković et al. reported reduction of AFB_1_ in cattle feed, by adsorption on copper-modified montmorillonite. This was possible due to the formation of a complex between the metal oxide-modified clay with the dicarbonyl group of AFB_1_.^70^ Likewise, ZnO ions initially adsorb rapidly to the vacant site of bentonite to give enhanced adsorption of AFs to the clay.^70^ However, chromophore-like Cur when added previously to the ZnOBt nanocomposite forms a chelate complex, which works as a mediation for rapid reaction with the analyte.^69^ AFs thus showed improved binding with electrochemically reduced ZnO NPs in the presence of oxidized Cur to cause a significant change in the color of the mixture as shown in our previous study.^55^ In our current investigation, we have studied that ZnO NPs incorporated bentonite nanocomposite when chelated at the diketone moiety of Cur showed improved electrostatic binding and stable color change in the presence of total AFs. Cur forms a chelate complex with ZnOBt mainly due to extended conjugation at the 1,3 diketone moiety as observed by FT-IR studies with lower wavelength shift in frequency of OH stretch for Zn(Cur)OBt in the presence of AFs. Addition of ZnOBt to Cur improved its ability to bind unsaturated aldehyde electrophiles of AFs. AFs in a highly alkaline medium share the electrostatic bond at an α,β-unsaturated ketone derivative with Zn(Cur)OBt and convert it to a highly polar medium with an improved hydrogen bond strength. Due to which, change in color of Zn(Cur)OBt from red (510 nm) to yellow (430 nm) in the presence of AFs was recorded by UV–Vis. This strategy significantly enhances the selectivity because of lesser chance of false positive results.^30,31,71^

As shown in Scheme 1, the addition of Cur at alkaline pH (9.54) to ZnOBt forms a red dispersed suspension. The enol form of Cur in non-polar and basic medium is prone to degradation. Hence, it stabilized in the presence of ZnOBt toward polar and neutral medium to its di-keto form. Under high basic pH conditions, aflatoxins are known to become unstable and sensitive; thus, addition of AFs in alkaline medium (9.54) to Zn(Cur)OBt suspension metabolizes AFs to coumaric acid or phenolate anions. AFs coumaric acid bind at its carbonyl group moiety to ZnO ions in the colored complex Zn(Cur)OBt and gradually change its color from red to reddish orange and yellow. The product formed is less deprotonated due to rapid oxidation of Cur and reduction in the pH from 9.54 to 7 by increasing AFs concentration from 0 to 20 ppb in food samples as shown in Scheme 1. This mechanism was studied and confirmed experimentally using UV–Vis, FT-IR, and NMR and theoretically using Job’s plot and Benesi–Hildebrand (B-H) equations.

#### 2.2.1 Colorimetric Binding by UV–Vis

**Figure 2:**
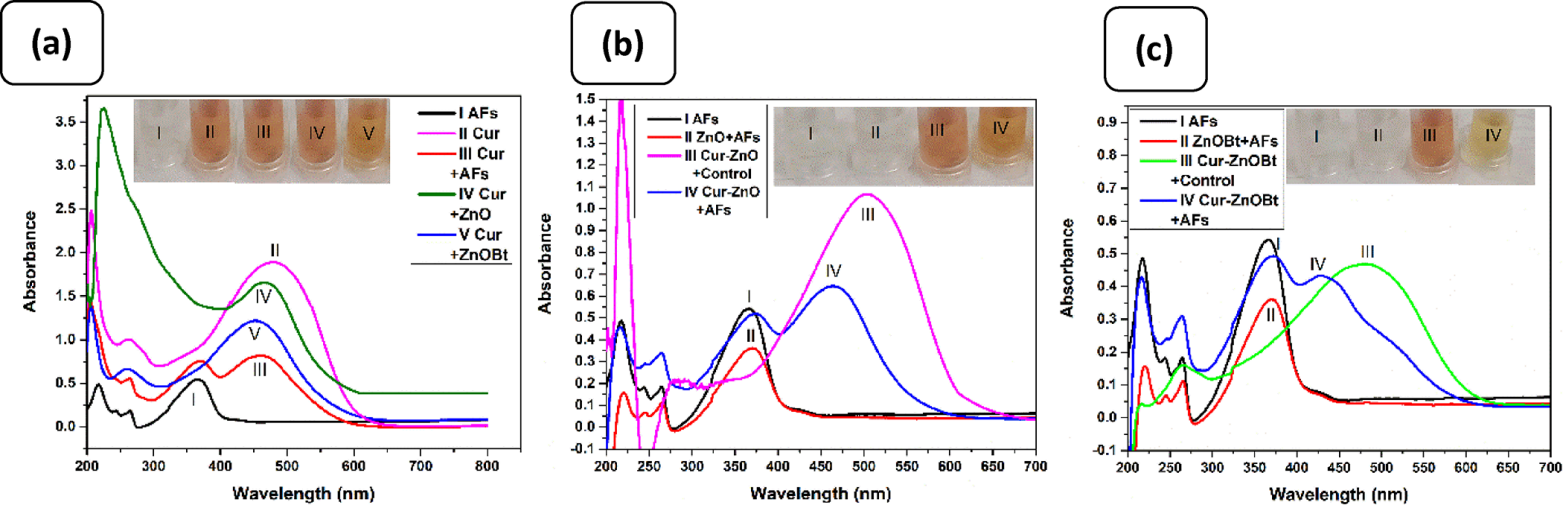
UV–Vis colorimetric binding of standard 5 ppm AFs (1 AFB_1_:0.1 AFB_2_:0.3 AFG_1_:0.03 AFG_2_) (I); (a) Cur (II), Cur+AFs (III), Zn(Cur)O (IV), Zn(Cur)OBt (V); (b) ZnO+AFs (II), Zn(Cur)O+control (III), Zn(Cur)O+AFs (IV); (c) UV–Vis spectra of ZnOBt+AFs (II), Zn(Cur)OBt+control (III), Zn(Cur)OBt+AFs (IV). Inset photographic images are the corresponding colorimetric response.

The colorimetric binding of Zn(Cur)OBt with increased AFs was studied by UV–Vis in the range of 200–800 nm. Initially, the Cur dye showed maximum absorption (λ_max_) in alkaline medium (pH 9.54) at 479 nm as shown in Figure 2a. Further, with addition of pure ZnO NPs and nanocomposite ZnOBt, Cur showed enhanced blue shift by 14 and 29 nm for Zn(Cur)O and Zn(Cur)OBt at λ_max_ 465 and 450 nm, respectively. This shift in absorption spectra could be due to oxidation of Cur with the ZnOBt nanocomposite and increased band gap between π–π∗ electronic transition from bonding to antibonding orbitals in Cur.^72^ It can be asserted that the formation of a mononuclear Zn(Cur)OBt complex occurred at the diketone moiety of Cur, which has improved optical and chemical stability with time as compared with complex Zn(Cur)O.^73^ Hence, suitably with addition of AFs to Zn(Cur)OBt causing a high energy difference, advance oxidation of dye Cur and improved electron transition between bonding and antibonding orbitals of Cur to cause a rapid color change from red to yellow. This is due to binding of AFs coumaric acid, which caused a decrease in pH of the composite and improved binding with Zn(Cur)OBt. The effect of solvent polarity in the absorption spectra of Zn(Cur)OBt was clearly visible upon UV irradiation as a negative solvatochromic effect. It can be seen from Figure 2a–c that there was no remarkable hypsochromic shift observed with only Cur and ZnO NPs in the presence of AFs in the solvent and in the control samples of both Zn(Cur)O and Zn(Cur)OBt complex at λ_max_ 503 nm. Meanwhile, with the addition of AFs, a remarkable blue shift of 39 and 73 nm was observed for AFs-Zn(Cur)O and AFs-Zn(Cur)OBt at λ_max_ 464 and 430 nm, respectively. Remarkably, ZnOBt showed improved binding for AFs in the complex than with bare ZnO NPs and can be used as a suitable substrate for total AFs binding in methanol with the food matrix.

#### 2.2.2 Functional and Molecular Binding by FT-IR

**Figure 3:**
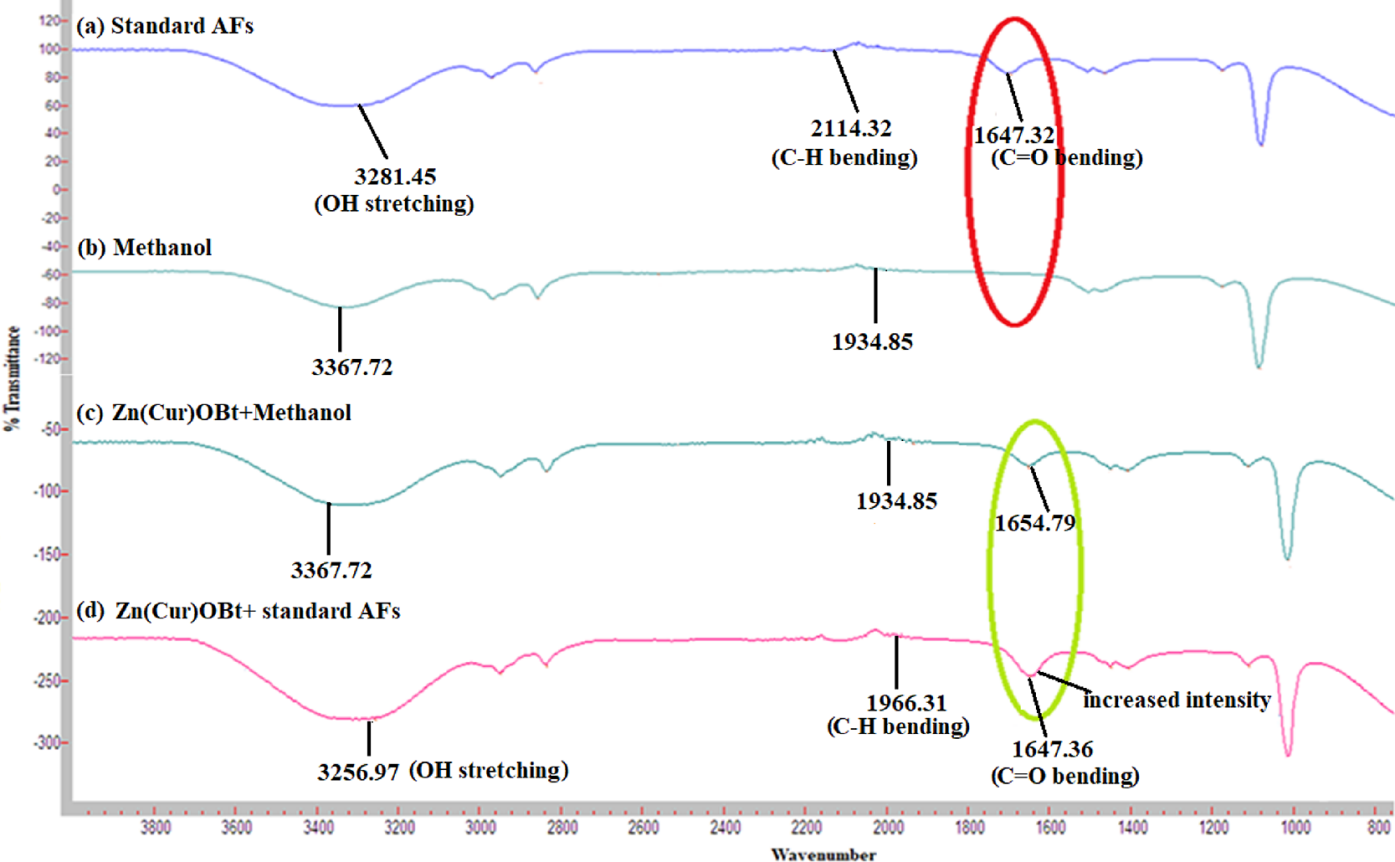
IR spectra of (a) standard AFs, (b) methanol, (c) Zn(Cur)OBt+methanol, and (d) Zn(Cur)OBt+standard AFs.

The molecular and functional interaction of Zn(Cur)OBt in the presence of AFs was studied by FT-IR. Figure 3 shows AFs molecular sites that bind the Zn(Cur)OBt composite possibly at 3256.974, 1966.615, and 1647.362 cm^–1^, indicating O–H stretching for carboxylic acid, C–H bending for alkane, and C=O stretching for conjugated ketone, respectively. Additionally, the fingerprint of two carbonyl oxygen atoms in AFs showed increased peak intensity in the test samples at 1647.362 cm^–1^ as studied by a previous report.^74^Figure 3 indicates the molecular site for standard pure AFs at nearby 3035–2821, 1770–1721, and 1570–1481 cm^–1^ spectral regions;^75^ for solvent methanol, at 3367.42 and 1934.85 cm^–1^;^76^ and for Zn(Cur)OBt-methanol (control), at around 1654 cm^–1^ corresponding to stretching vibrations of ν(C=C) and ν(C=O) in the Cur–Zn(II) interaction complex, respectively;^77^ ν(=C) and ν(C=O) were red shifted to 1647.36 cm^–1^ due to Zn(Cur)OBt-AFs interaction. Additionally, a blue shift in OH stretching and red shift in C–H bending are seen for Zn(Cur)OBt-AFs complex at 3256 and 1966 cm^–1^, respectively, indicating the presence of AFs in the composite. The π systems like phenyl groups in Zn(Cur)Bt in the presence of AFs showed an increase in hydrogen bond strength by conjugation with the enol group. The phenolic group n(OH) vibrations showed the frequency region in FT-IR at 3367 cm^–1^ in methanol, but in the presence of AFs, it is shifted to lower frequencies at 3256 cm^–1^. This could be due to the formation of intermolecular and intramolecular hydrogen bonding in the Cur molecule in the composite.

#### 2.2.3 Nuclear Magnetic Resonance (NMR)

**Figure 4:**
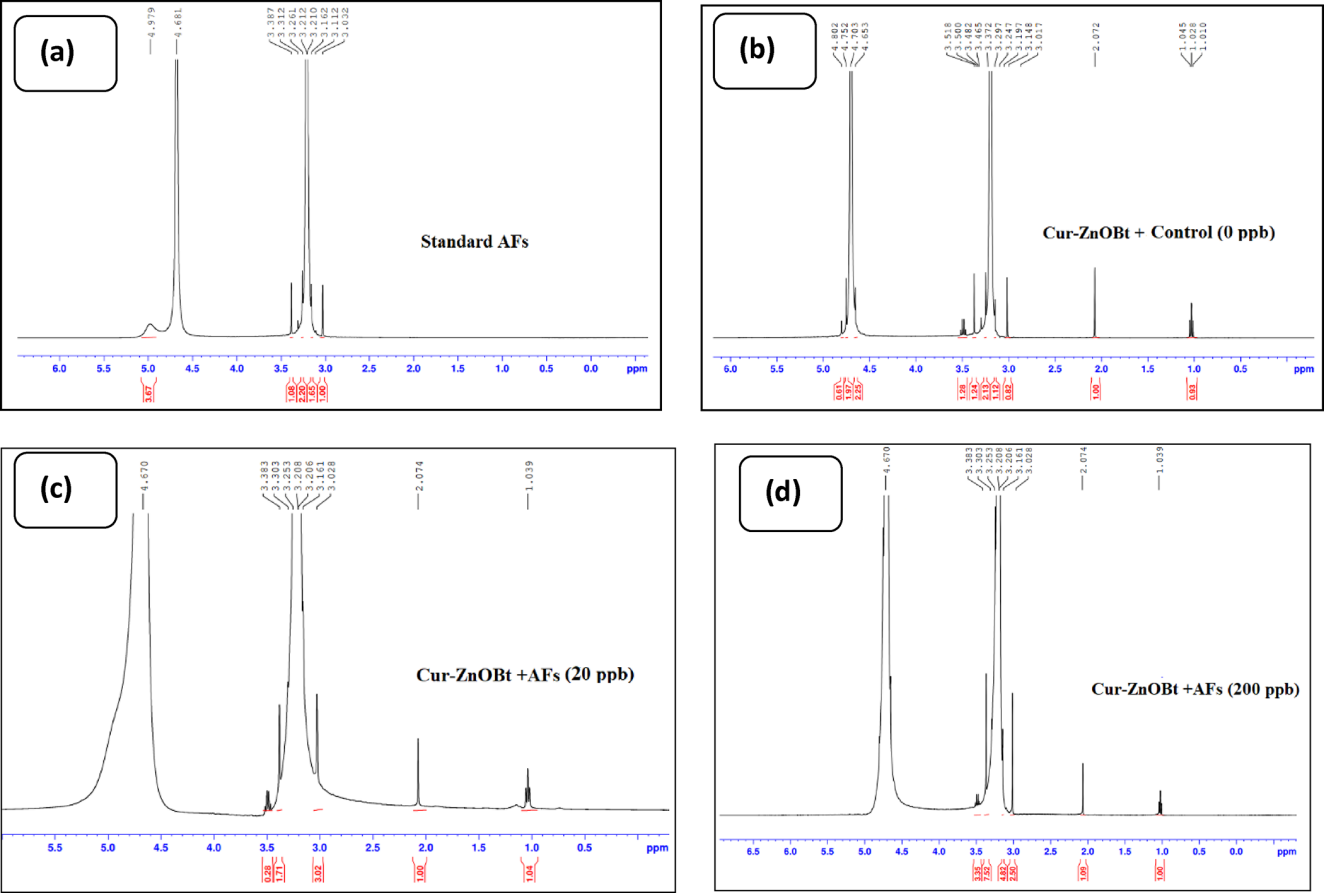
^1^H NMR chemical shift (δ) in ppm for (a) standard AFs (AFB1, AFB2, AFG1, and AFG2), (b) Zn(Cur)OBt+Control, and (c) Zn(Cur)OBt-AFs at 20 ppb and (d) 200 ppb.

Proton nuclear magnetic resonance (NMR) spectroscopy is a powerful tool for chemical profiling, also known as spectral fingerprinting, for its inherent reproducibility. In this study, we analyzed the binding mode of Zn(Cur)OBt with coumaric acid of AFs by ^1^H NMR. As shown in Figure 4, in the absence of AFs, the Zn(Cur)OBt+Control displayed the chemical shift (δ) region between 4.4 and 6.0 ppm to represent aromatic ether protons, which are not found in the presence of AFs at 20 and 200 ppb due to stable polyphenol AFs-Zn(Cur)OBt formation. The number of protons at the aliphatic region^79^ between 0 and 4.4 increases as AFs concentration increased with Zn(Cur)OBt due to gain of proton from the AFs coumaric acid group. Table 2 shows the ^1^H chemical shift alignment and % distribution in standard AFs, control, and test samples for Zn(Cur)OBt at a AFs concentration of 20 and 200 ppb, respectively. The results are in accordance with UV–vis spectroscopy as the absorption maximum of fully deprotonated (red in color) Zn(Cur)OBt in alkaline pH (pH 9.54) is at 450 nm. The p*K*_a_ in the pH range of 9.54–7.5 changes Cur in the Zn(Cur)OBt complex with an increase in AFs concentration from red to yellow. The chemical reactivity and solubility of the anionic Cur, i.e., in the neutral pH range, decrease, and this form of Cur is less water soluble than the basic form.^78^ Thus, an increase in the number of protons observed at the aliphatic region with increased AFs was indicative of AFs proton addition to Zn(Cur)OBt. The cross-peaks indicated that Zn(Cur)OBt interacts with the carbonyl group of AFs coumaric acid.^77^ Interestingly, aromatic ether protons of Cur showed clear cross-peaks in the mononuclear Zn(Cur)OBt complex in the control sample but are not found in Zn(Cur)OBt-AFs at 50 and 200 ppb. Thus, it indicated that the total number of protons increased with increased AFs.

**Table 2:** % Distribution of ^1^H Based on ^1^H NMR Analysis of Standard AFs and Zn(Cur)OBt with Three AFs Concentration (0, 20, and 200 ppb)^a^

^1^H chemical shift range (δ) (ppm)	proton assignment	samples	% distribution
0–4.4	aliphatic	(1) standard AFs: 3.002, 3.112, 3.162, 3.210, 3.212, 3.261, 3.312, 3.387	5
		(2) Zn(Cur)OBt+Control (AFs 0 ppb): 1.010, 1.028, 1.045, 2.072, 3.017, 3.148, 3.198, 3.297, 3.372, 3.465, 3.482, 3.50, 3.518	8
		(3) Zn(Cur)OBt-AFs: (AFs 20 ppb): 1.039, 2.074, 3.028, 3.161, 3.206, 3.208, 3.253, 3.303, 3.383	8
		(4) Zn(Cur)OBt-AFs: (AFs 200 ppb): 1.039, 2.074, 3.028, 3.161, 3.206, 3.208, 3.253, 3.303, 3.383	20
4.4–6	ether	(1) Standard AFs: 4.681, 4.979	0
		(2) Zn(Cur)OBt+Control: 4.653, 4.703, 4.752, 4.802	5
		(3) Zn(Cur)OBt-AFs: (20 and 200 ppb): 4.670	0
		(3) Zn(Cur)OBt-AFs: (200 ppb): 4.670	0

a The total number of protons increased in Zn(Cur)OBt-AFs with increased AFs.

#### 2.2.4 Benesi–Hildebrand (B-H) Plot

**Figure 5:**
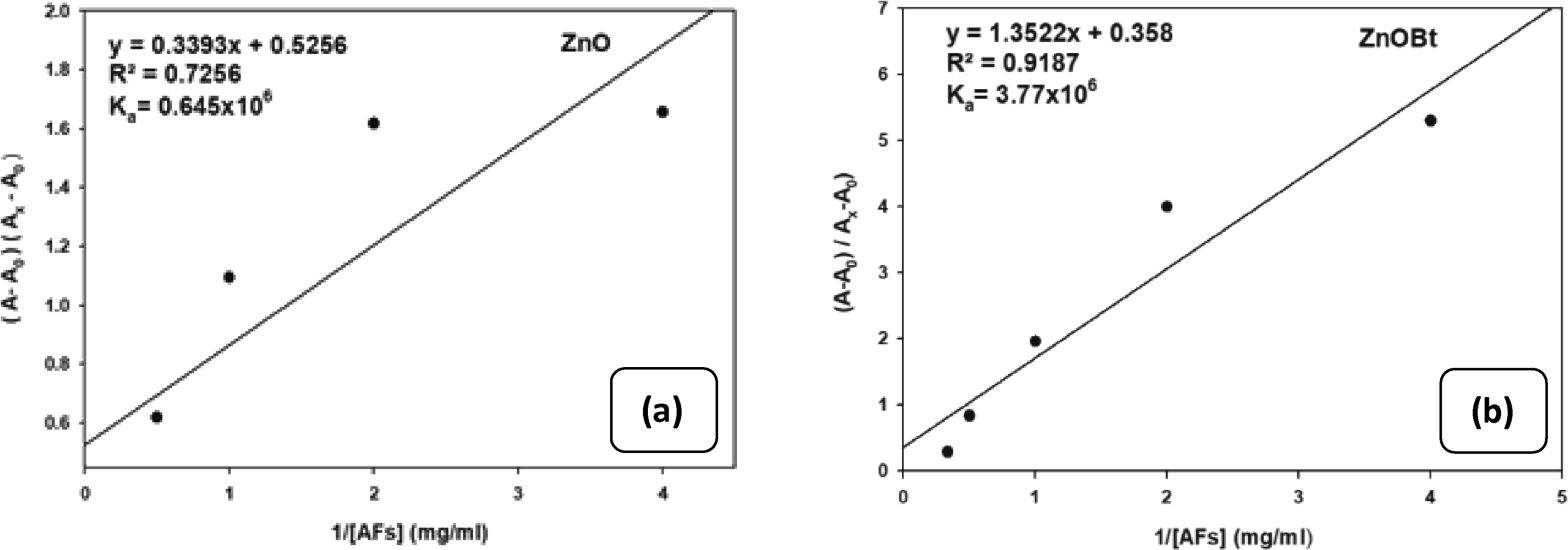
Benesi–Hildebrand equation plot from the absorption titration values at 367 nm for AFs in the presence of (a) Zn(Cur)O and (b) Zn(Cur)OBt.

The Benesi–Hildebrand method is a statistical method in physical chemistry used to define the equilibrium constant *K*_a_ as well as the stoichiometry of non-bonding molecular interactions. It is used for defining a one-on-one charge-transfer complex or host–guest complex interaction. Theoretically, it is explained as, with the assumption that one of the reactants is present in excess of the other, thus the absorption spectra of the characteristic reactant are transparent for collective absorption of the reaction system. Hence, by computing the absorption before and after the formation of the product at its equilibrium, the association constant value is determined. In Figure 5, the one-on-one interaction between the substrate Zn(Cur)O and Zn(Cur)OBt with the analyte (AFs) by simple UV–Vis is described. The core of this plot includes computing the acquired absorbance of dispersed ZnO and ZnOBt with AFs wherein concentration units play a critical role for the characteristic complex formed using B-H. Thus, the entire B-H plot is used to compute the binding constant *K*_a_ value to determine the concentration scale, which binds correctly at simple equilibrium. By plotting the ratio of the absorbance intensity with respect to reciprocal concentration of AFs, the Benesi–Hildebrand association constant for the complex formation (*K*_a_) was calculated from the ratio of the slope to the intercept. The *K*_a_ value of Cur with AFs in the presence of ZnOBt and ZnO was found to be 3.77 × 10^6^ and 0.644 × 10^6^ mol^–1^ L as shown in Figure 5a,b. The higher value of the binding constant indicates the strong interaction between ZnOBt and AFs. Thus, in the presence of AFs, rapid color change and oxidation of dye Cur are observed more with ZnOBt than with bare ZnO NPs.

#### 2.2.5 Binding Ratio by the Job Plot Method

**Figure 6:**
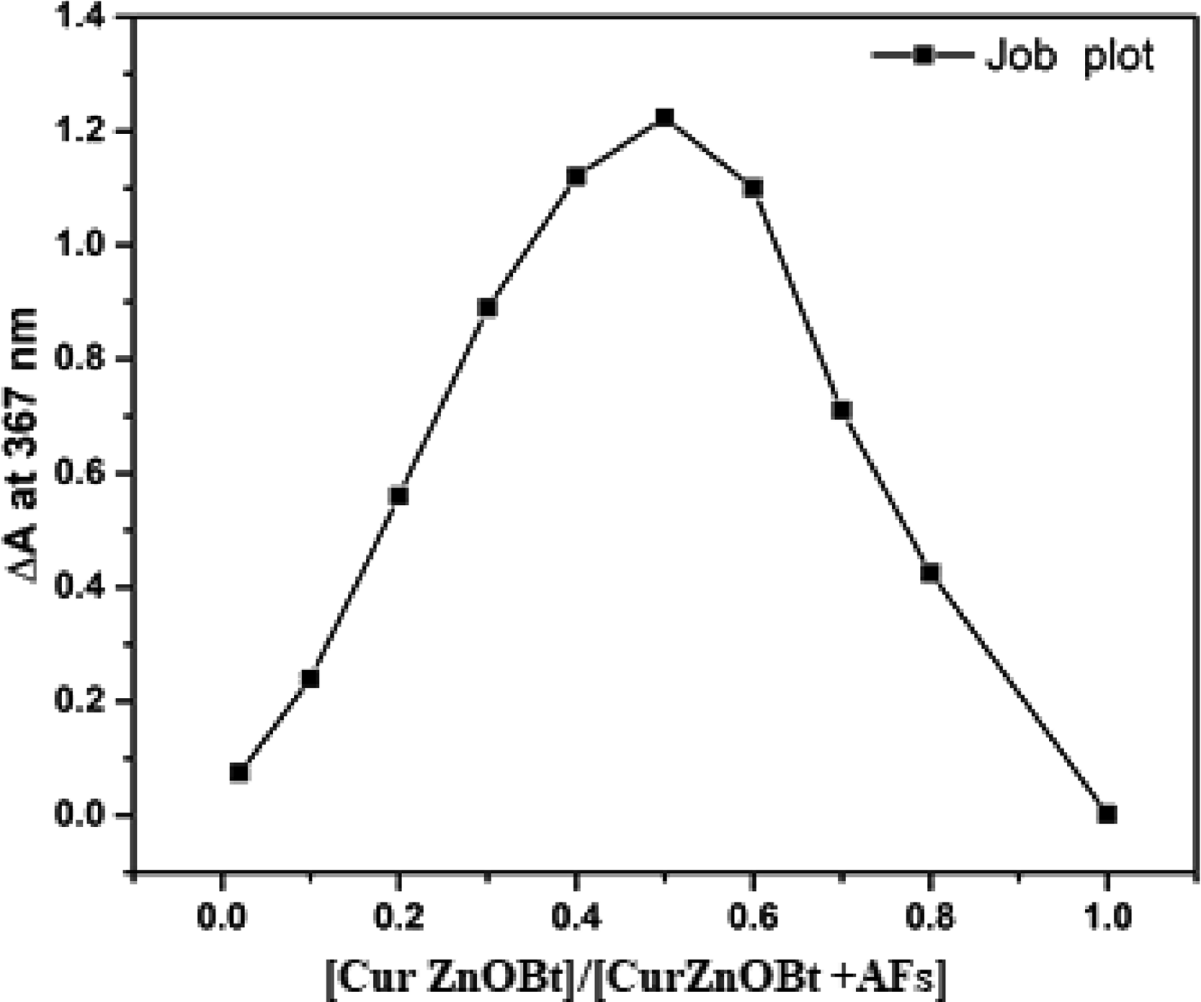
Job’s plot for Zn(Cur)OBt with AFs. The concentration of ZnOBt is kept constant for ZnOBt+AFs at 0.5 mg/mL. Δ*A* indicates the difference between the absorbance before and after addition of AFs.

A Job plot is known as continuous variation method used to define the stoichiometric ratio of a binding event. The maximum and minimum points in a Job plot correspond to the stoichiometric ratio of the binding reactants. It provide an insight into the equilibrium constant (*K*_eq_) of the product formation. More curvature in a Job plot represents the even distribution of equilibrium, while a triangular plot indicates a large value of *K*_eq_. To determine *K*_eq_, the ratio of analytes is established in a solution. In Figure 6, the binding stoichiometry ratio of Cur and AFs in the presence of ZnOBt was plotted as the difference (Δ*A*) between the absorbance at 367 nm before and after addition of AFs versus function of the mole fraction of Zn(Cur)OBt under a constant total concentration. The peak of the Job’s plot obtained correspond to the mole fraction of Zn(Cur)OBt bound to AFs. The Δ*A* value reached a maximum when the mole fraction was 0.5, indicating that the complex of Zn(Cur)OBt and AFs formed a 1:1 stoichiometric ratio. Thus, analysis of absorbance data in terms of Job’s plots and the Benesi–Hildebrand equation is indicative for the efficient formation of a stable 1:1 complex due to the favorable electrostatic bond interaction between the carbonyl group of AFs and Zn ions bound with the Zn(Cur)OBt complex. Thus, Zn(Cur)OBt binds efficiently with AFs established by Job’s plot method.

### 2.3 Optimization of Sensor Studies

To optimize the colorimetric sensor, working conditions were investigated with the following factors: concentrations of (a) ZnOBt,(b) NaOH, and (c) curcumin and (d) incubation time and pH stability. Corresponding figures (Figures S1–S4) and data are shown in the Supporting Information. The test results indicated the best possible values at (a) 0.25 mL (of 0.5 mg ZnOBt/mL dispersion in DW); (b) 0.1 M NaOH; (c) 0.166 mL (of 1 mg curcumin/mL solution in ethanol); and (d) 2 min as total incubation time and pH 9.54 for higher accuracy and stability of results. Food samples were collected from local/commercial markets for comparative analysis of the Zn(Cur)OBt sensor with the standard LC/MS-MS technique to calculate the accuracy. Alike values were known between the two sensing modes as shown in Figure S5a. The biosensor showed optimal reproducible signals with related standard deviation (RSD) achieved between 5.6% and 6.9% for three repetitive measurements of AFs concentrations. The reproducibility was thus confirmed at all stages of the reaction. The stability of the experiments was checked at room temperature (RT, 25 °C) and investigated for its function with respective 9 weeks at different time intervals. The measurement obtained a consistent signal with time (Figure S5b), with minimal variation for 9 weeks obtained to be 6%. This showed the stability of the Zn(Cur)OBt reactions platform as 94%, which is optimal for food sample analysis. Adding to it, the relative deviation of Zn(Cur)OBt with the standard LC/MS-MS method was limited maximally to 14% over various samples to acquire a minimum accuracy of 86% as shown in Table S1.

### 2.4 Interference Study of the Sensor with Mycotoxins

**Figure 7:**
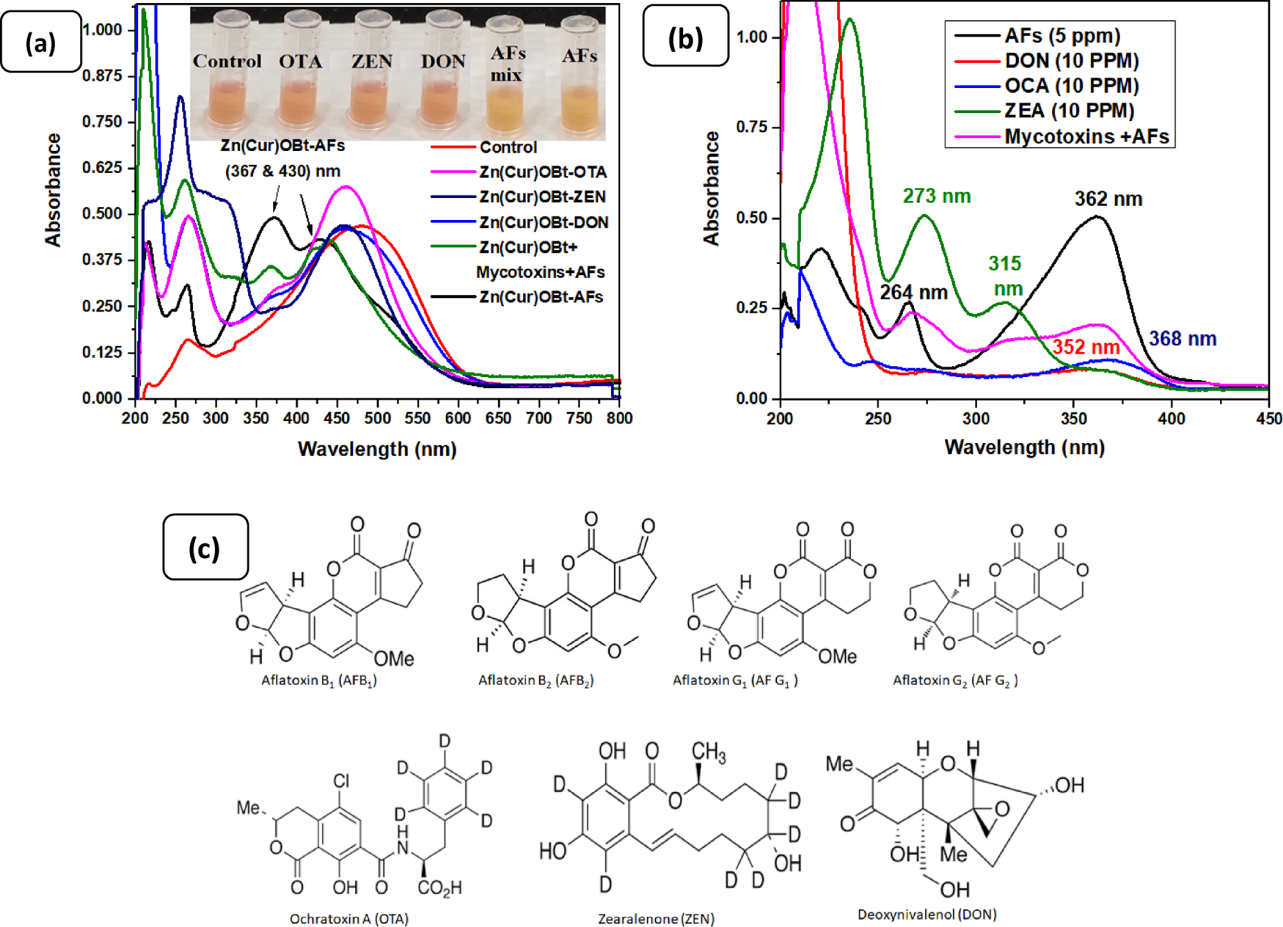
Selectivity of Zn(Cur)OBt-based colorimetric detection assay. (a, b) Absorption spectra of Zn(Cur)OBt mixed solutions in the presence of other related toxins; inset shows the corresponding visual observation of Zn(Cur)OBt mixed solutions in the presence of other related toxins. (c) Molecular structure of standard toxins.

The selectivity of analytical reports submitted previously for AFs reported usual testing of the method with other mycotoxins species. In our test, we have chosen three kinds of mycotoxins (OTA, ZEN, and DON), mostly affecting corn and almond crops.^80,81^ Further, we studied their colorimetric behavior to entail the selectivity of the current method. For improved inter-assay and intra-assay comparison, mycotoxins (10 ppm each) and total AFs (5 ppm) were tested for standard pure solution and mixed mycotoxins, respectively. Figure 7a highlights that various other mycotoxins recorded similar responses as the blank sample, showing no interference with total AFs in inter- and intra-assay selectivity. The results showed that the OTA, ZEN, and DON standard solutions showed a red color with no observed peak at 430 nm, implying that they do not bind with ZnOBt. Further, OTA, ZEN, and DON in the presence of total AFs showed an orange color at the absorption peak of 430 nm, respectively. It indicates that only AFs can bind selectively to ZnOBt in the reaction composite, irrespective of any interference caused by other mycotoxins or the blank sample. This was evident because OTA, ZEN, and DON in their chemical structures contained no adjacent carbonyl group or lactone moiety as apparently seen in the structure of all primary AFs (Figure 7d). A similar pattern of color change with Zn(Cur)OBt for AFs was found in test and control samples of corn and almond food samples, as noticed by the L*a*b* value of a hand colorimeter as shown in Table S2, Supporting Information. Thus, Zn(Cur)OBt as a core sensing material demonstrated an excellent selectivity for AFs detection.

### 2.5 Sensitivity of the Sensor

**Figure 8:**
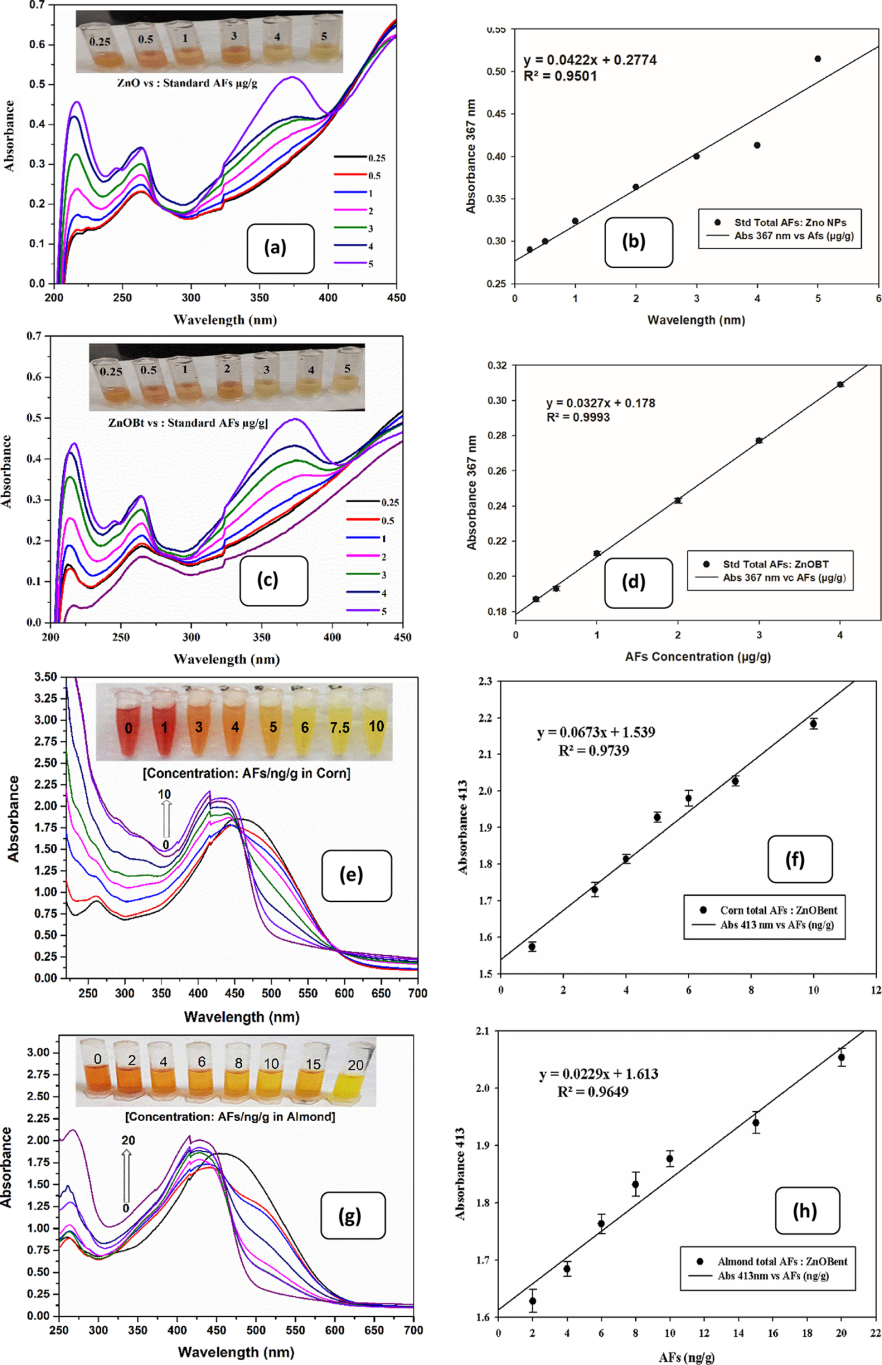
(a, c) UV–Vis spectra of ZnO NPs and ZnOBt containing 1 mg/mL Cur in the presence of AFs with different concentrations. The inset portion shows the corresponding images of reaction. (b, d) Absorption at 367 nm of ZnO NPs and ZnOBt versus the concentrations of AFs (error bars represent the standard deviation of three data values). (e, g) UV–Vis spectra of Zn(Cur)OBt in the presence of AFs spiked corn and almond. (f, h) Absorption at 413 nm of Zn(Cur)OBt versus the concentrations of AFs spiked corn and almond.

To analyze the sensitivity of the sensor and compare ZnO with ZnOBt, a series of different AFs concentration in the proportions of 1.0:0.1:0.3:0.03^80^ were studied for the linear range from 0.25 to 5 μg/g. The increased AFs value caused a systematic increase in absorbance value and color change at the characteristic peak for standard AFs (367 nm) in ZnOBt (*R*^2^ = 0.99) than bare ZnO NPs (*R*^2^ = 0.95) as shown in Figure 8a,b. Further, ZnOBt are studied for spectroscopic analysis to detect total AFs in almond kernel ground powder and corn flour as shown in Figure 8c,d. The linear correlation existed for increased AFs in the linear range of 0.5–10 ppb in corn with *R*^2^ = 0.9739 (Figure 8e,f) and 0.5–20 ppb in almond with *R*^2^ = 0.9649 (Figure 8g,h). The AFs detection limit (3σ) for respective food categories was calculated as 2.74 ppb for corn and 4.37 ppb for almond, which is found to be lesser than the maximum residual level (MRL) defined by India (30 ppb), China (5 to 20 ppb), U.S. (20 ppb), and EFSA (8 or 10 ppb).^5−8^ Essentially, the method can quantify total AFs without prior use of an antibody, aptamer, enzyme, or any other expensive screening techniques. Table 3 shows a comparative view of our sensor studied in corn and almond with respect to conventional and rapid biosensors. It indicated that the current study has the potential to sensitively and selectively detect total AFs together in corn and almond at LOD values complying with the regulatory standards. Hence, it can be an interesting option to replace traditional complicated, time-consuming, tedious, and expensive techniques at the small scale for pre-processing, post-harvest processing, and storage at the industrial scale for corn and almond, respectively.

**Table 3:** Comparative List of Selective Biosensor with Current AFs Method in Foods

methods	food matrix	linear range (ng/mL)	detection limit (ng/mL)	selectivity	the use of antibody	reference
enzyme-linked immunosorbent assay	peanut	0.12–103	0.05	AFB_1_ and G_1_	yes	(11)
chemiluminescence-based immunoassay	rice, mung beans	0.003–0.03	0.0015	AFB_1_	yes	(14)
lateral flow immunoassay	pig feed		5	AFB_1_	yes	(13)
fluorescence immunoassays	peanut	0.01–5	0.008	AFB_1_	yes	(16)
immunochip	drinking water	0.04–1.69	0.01	AFB_1_ and M_1_	yes	(15)
membrane-based assays	cereal sample		20	AFB_1_	yes	(12)
immunosensor (Cur-ZnOBt)	corn	0.05–200	0.02	AFB_1_	yes	(17)
	corn	1–10	2.74	AFs	no	this study
	almond	1–20	4.37	AFs	no	this study

### 2.6 Validation by Standard LC/MS-MS

Cereals and tree nuts are widely consumed and mostly contaminated with AFs.^80^ To study the applicability of the colorimetric detection in corn and almond, validation studies were performed with the standard LC/MS-MS method for AFs addition and recovery experiments. In our study, corn and almond were determined as AFs-free (Figure S6, Supporting Information) and were spiked with total AFs. As described in Table 4, the results hold good correlation between the spiked and recovered value in visual by the developed Zn(Cur)OBt-based colorimetric sensor and standard LC/MS-MS method. The mean recovery calculated as 89.4–97.7% held acceptable RSD values, less than 8.1% for the colorimetric method.

**Table 4:** Recovery Validation Studies of Total AFs by the Developed Colorimetric Sensor and LC/MS-MS for Corn and Almond Samples (*n* = 5)

		developed colorimetric nanosensor (visual method)			LC-MS/MS		
food samples	spiked AFs level (μg/kg)	mean ± SD	recovery (%)	RSD (%)	mean ± SD	recovery (%)	RSD (%)
corn sample	5	4.82 ± 0.2	96.4	4.1	4.2 ± 0.2	83.3	4.3
	15	13.7 ± 1.1	91.2	8.1	12.7 ± 0.6	84.5	5
	30	26.8 ± 1.8	89.4	6.6	25.1 ± 1.7	83.5	6.9
almond sample	5	4.7 ± 0.4	94.1	8.6	4.2 ± 0.2	83.3	4.3
	15	13.3 ± 1.3	83.2	9.6	12.7 ± 0.6	84.5	5
	30	27 ± 1.6	90.2	6	25.1 ± 1.7	83.5	6.9

Thus, we present a simple method of AFs label-free color-based detection by UV–Vis. It does not include any use of high-grade AFs-selective antibody, aptamer, DNA-enzyme, and chemical dye for colorimetric results. It showed visible detection of all aflatoxins (B_1_, B_2_, G_1_, and G_2_) in corn and almond food products based on the cost-effective and environmentally friendly metal oxide nanoclay composite and organic dye curcumin. This is the first method reported based on the zinc oxide-bentonite clay nanocomposite for total AFs detection in corn and almond.

## 3 Conclusions

A simple rapid label-free method using the ZnOBt nanocomposite is developed for total AFs detection in corn and almond. ZnOBt showed the improved morphological surface area and optical band gap of ZnOBt than ZnO NPs. The AFs presence in the Zn(Cur)OBt complex showed improved binding as compared with bare Zn(Cur)O, causing rapid color change from red (503 nm) to yellow (430 nm). The AFs-Zn(Cur)OBt binding is characterized by ^1^H NMR, indicating the improved alkaline proton in the complex at the 0–4.4 region with increased AFs concentration. UV–Vis showed AFs binding to Zn(Cur)OBt at 367 nm, and FT-IR indicated binding at 1647 cm^–1^ for the carbonyl group of AFs coumaric acid. The B-H plot and Job plot also indicated theoretically that binding of the ZnOBt nanocomposite for AFs increased in a 1:1 stoichiometric ratio. This is the first report for colorimetric detection of total AFs in corn and almond food crops at a LOD value of 2.74 and 4.37 ppb, respectively. However, for large complex food matrices, it may require a pretreatment sample clean up technique for remediating interferences. Hence, working on its improved sensing system for total AFs detection will be our future endeavor with different agro-products and nanocomposites.

## 4 Experimental Section

### 4.1 Reagents

All chemicals and reagents were of analytical grade and utilized without additional purification. Aflatoxin B_1_ (AFB_1_), aflatoxin G_1_ (AFG_1_), aflatoxin G_2_ (AFG_2_), aflatoxin B_2_ (AFB_2_), zearalenone (ZEN), deoxynivalenol (DON), ochratoxin A (OTA), methanol, Cur, zinc acetate dehydrate, *N*,*N*-dimethylformamide (DMF), boric acid, and sodium hydroxide were purchased from Merck Sigma-Aldrich Pvt. Ltd., USA. The AFs and other mycotoxins mother stocks are kept in an amber flask in a closed and refrigerated chamber at −20 °C. Bentonite (aluminum silicate hydrate montmorillonite) was purchased from SRL Pvt. Ltd., India. Ultrapure water (18.2 MΩ·cm) utilized in the experiment was obtained from a Milli-Q purification system (Millipore), and NaOH, boric acid, and ZnOBt dispersion were made with water. The respective standard solution of mycotoxins and individual aflatoxins (in the ratio of 1.0:0.1:0.3:0.03) were mixed in methanol at a ratio of 50 μg mL^–1^. The required working solution was prepared with a methanol–water solvent in a ratio of 3/7 (V/V).

### 4.2 Instrumentation

The surface-modified functional groups of nanocomposites and binding mechanism of AFB_1_ with Zn(Cur)OBt weres explained by FT-IR (Agilent Technologies Cary 630 FT-IR) having measured at a 8 cm^–1^ resolution with a 1 min collection time and 16 scans. Meanwhile, the optical absorption spectra of nanocomposites and colorimetric analysis for AFs were analyzed by UV spectroscopy (Shimadzu UV-2600) with a 10 mm path length fused-silica cuvette at room temperature. The crystallinity and size distribution of the nanocomposite were determined by XRD (Ultima IV X-ray diffractometer), and the morphology was examined by SEM (ZEISIS-EVO 18 special edition). NMR analyses were recorded on a Varian Unity Inova spectrometer at a resonance frequency of 399.961 MHz for ^1^H using a 5 mm pulsed field gradient indirect detection probe or a 10 mm broadband probe. ^1^H spectra were obtained from samples dissolved in D_2_O_2_. The solvent signals (D_2_O_2_^1^H 4.7 ppm) were used as the internal reference. The samples were extracted in D_2_O_2_ solvent, and the extract was investigated for ^1^H NMR spectroscopy. Subsequently, the spectra were integrated over spectral regions to quantify classes of hydrogen atoms in the complex and characterized the chemical bond formation and semi-quantitative estimation for different AFs concentration bound with Zn(Cur)OBt at concentration. The total color difference and the Commission Internationale de l’Eclairage (CIE) L*a*b* coordinate values for colorimetric analysis were studied using a hand colorimeter (CR 400, Konica Minolta, Japan).

### 4.3 Synthesis of ZnOBt

Addition of ZnO to bentonite clay was prepared by a quick and simple alkaline ion exchange method. Bentonite was submitted to an ion-exchange process mainly by direct intercalation of ZnO NPs to in situ clay interlayers. To prepare the hybrid material, 10 g of purified sodium bentonite was suspended with 2 g of zinc acetate dehydrate in 250 mL of DMF and was further sonicated for 3 h to get a homogeneous dispersion. To this mixture, 100 mL of NaOH/H_2_O (0.1 M solution) was stirred constantly for 1 h. After centrifugation, the nanocomposite obtained was dispersed in alcohol and dried at 75 °C under vacuum for 4 h followed by calcination at 200 °C for 2–3 h.^31^ The obtained dried ZnOBt was dispersed and found stable in Milli-Q water (0.5 mg/mL). Cur-functionalized ZnOBt was prepared by adding 0.25 mL of ZnOBt dispersed solution to 0.5 mL of curcumin. Zn(cur)OBt was found to be stable in methanol at pH 9.54 for AFs colorimetric reaction.

### 4.4 Colorimetric Detection of AFs

In a typical experiment, 3 mL of NaOH (0.1 M), 1 mL of boric acid (0.1 M), and 2 mL of Cur (1:1 mg/mL in ethanol) were added to a cleaned, dried test tube and vortexed for 2 min. To 0.5 mL of the above mixture, 3 mL of methanol was added. Then, we took 0.1 mL of the mixture (for standard reaction) and 0.5 mL of the mixture in a separate test tube (for food sample reaction) and added 0.25 mL of ZnOBt and 0.5 mL of AFs test sample (for both the standard and food). Further, we incubated it at RT for 2 min and analyze it for UV–Vis, FT-IR, and NMR colorimetric studies.

### 4.5 Pretreatment of Food Samples

The sample pretreatment for AFs analysis was done as per the standard method given by the international organizations such as FAO/WHO.^80,82,83^ Concisely, to 10 g of the ground food matrix was added 25 mL of methanol/water in a ratio of 8/2 (V/V) and stirred for 3 min. Post-centrifugation at 600 rpm for 5 min, the supernatant (10 mL) was partitioned in a 50 mL centrifuge tube with *n*-hexane (6 mL) and vortexed for a minute. After partitioning of the phases post-centrifuge (6000 rpm, 5 min), the upper phase including *n*-hexane was removed and added with 10 mL of chloroform to the solution. The obtained mixture was mixed in a vortex for a minute and centrifuged (6000 rpm, 5 min). It is followed by removal of the upper phase, and chloroform was evaporated with a nitrogen blow. The residue obtained was made up to 1 mL with methanol/water and prepared in a ratio of 3/7 (V/V) for the analysis by the developed colorimetric setup.

### 4.6 Determination of the Binding Constant (*K*_a_) by the B-H Plot and Stoichiometry of Metal–Ligand Complexes by Job’s Plot Experiment

The binding constant (*K*_a_) was determined from the Benesi–Hildebrand equation^84,85^ as follows:
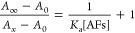
1where *A*_∞_ and *A_x_* are the absorbances in the presence of AFs at saturation and an intermediate concentration, respectively, and *A*_0_ is the absorbance of Cur-ZnOBt in the absence of AFs. *K*_a_ could be calculated from the slope of (*A*_∞_ – *A*_0_)/(*A_x_* – *A*_0_) against 1/[AFs].

Meanwhile, the continuous variation method, known as the Job plot,^85^ is the most commonly applied method for the determination of stoichiometry of very stable metal–ligand complex chemical entities. It is assumed that the H*_n_*G*_m_* complex is the only one formed and therefore the only one giving rise to Δ*Y*, where H is the host or reaction material, G is the guest or analyte, and Δ*Y* is a set of *Y* values obtained gives a titration curve that is a plot of *Y* (or Δ*Y* = *Y* – *Y*_0_) versus [G]_0_.

### 4.7 Calculation of Stability, Precision, and Accuracy

To obtain efficient results and for generation of automated data calculation of identified studied samples, it is essential to adapt an approach that establishes the stability, accuracy, and reproducibility of measurements. Stability was determined as variation in a detected signal over a period of time or storage. Figure S5b denotes the baseline value of AFs determined by an average of detected signal each week. Further, to calculate the stability value, the RD of the detected signals with respect to the baseline was deduced by following eq 2.
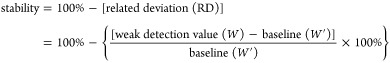
2

Precision or reproducibility was determined using a coefficient of variation (CV; eq 3) at each stage after addition of the reaction composite and AFs to get a consistent reaction and production of detected signals.
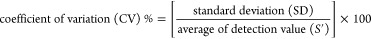
3

Accuracy describes the variation between biosensor-detected values and the actual values. In tests of unknown samples, realistic values were determined and compared by the gold standard LC/MS-MS method. The accuracy points for AFs detection was determined by eq 4.
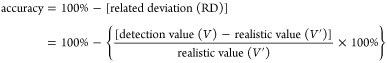
4

## Supporting Information Available

Label-free colorimetric sensor based on curcumin-functionalized Zn-bentonite nanocomposite for total aflatoxins detection in corn and almond (PDF)
