# Supplementary material for: Sensitive Metal Oxide-Clay Nanocomposite Colorimetric Sensor Development for Aflatoxin Detection in Foods: Corn and Almond
Source: ACS Omega. 2021 Jun 3;6(23):14911–25. doi: 10.1021/acsomega.1c00750 (PMC8209818; doi:10.1021/acsomega.1c00750)
Supplement: Supplementary file 1 — ao1c00750_si_001.pdf [file ao1c00750_si_001.pdf]

Supplementary material

Sensitive metal oxide-clay nanocomposite colorimetric sensor development for aflatoxin detection in food: corn and almond

Nishtha Khansili[a], Prayaga Murali Krishna[a, *]

[a] Department of Basic and Applied Science, National Institute of Food Technology Entrepreneurship and Management (NIFTEM), Haryana 131028, India

* Corresponding author: physicsres.niftem@gmail.com (Prayaga Murali Krishna)

Optimization of experimental conditions

(a) The concentration of ZnOBt

The concentration of zinc bentonite has significant role on the binding of AFs derived phenolate anions [1, 2] to the composite Cur- ZnBt. Thus, the concentration of ZnBt was optimized in the visual method. As described above Cur- ZnBt -AF has absorption band at 367 nm. So the increase in absorbance at 367 nm was calculated to know the degree of AFs binding to the composite. A higher absorbance indicates higher binding. The concentration of ZnBt was optimized from 0.1 mg/ml to 1 mg/ml, the plot of absorbance at 367 nm versus the ZnBt concentration in the suspension was calculated as shown in Supplementary material (Figure S1). The A_{367} value increased sharply in the concentration of Zn-Bt range from 0.1 mg/ml to 0.45 mg/ml and reached a maximum when the concentration is 0.478 mg/ml. Thus, 0.5 mg/ml ZnBt that ensured improved binding strong enough in the composite ZnBt-Cur was selected in our experiment.

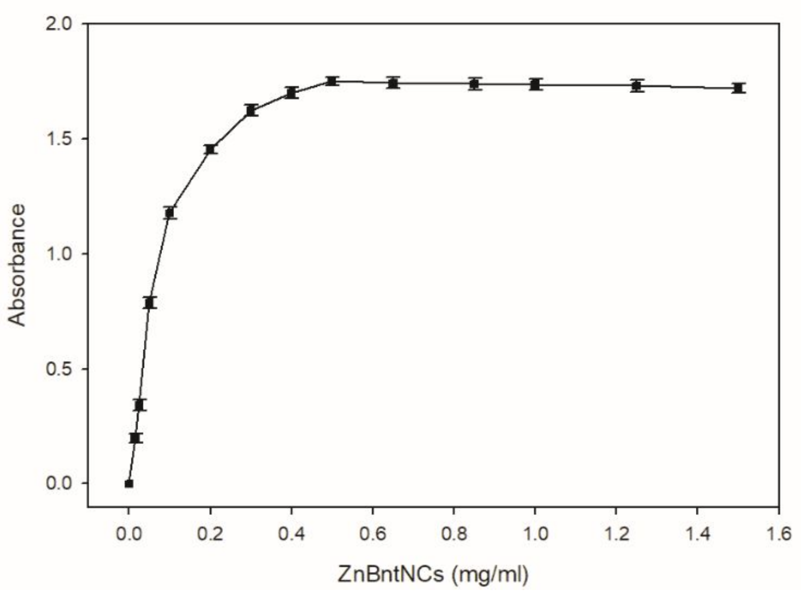

Figure S1. Effect of modified concentration of ZnBt on the binding of AFs phenolate anions to ZnBt-Cur composite.

(b) Concentration of curcumin

To achieve the best sensing performance, the concentration of curcumin was further standardized. Figure S2 shows the photographic image of Cur-ZnBt in the presence of AFs at different concentration. It is evident that ZnBt does not alone bind to AFs if the concentration of Cur drops below 0.5 mg/ml. On the other hand, binding reached maxium if the concentration is increased to 1 mg/ml or higher. This is accompanied by color change from red to orange. However, by decreasing the amount of curcumin, sensitivity of the method will be increased. In other words, high concentration of curcumin consumes more AFs and have an adverse effect on sensitivity. Therefore, the concentration of curcumin at 1 mg/ml was chosen in our study.

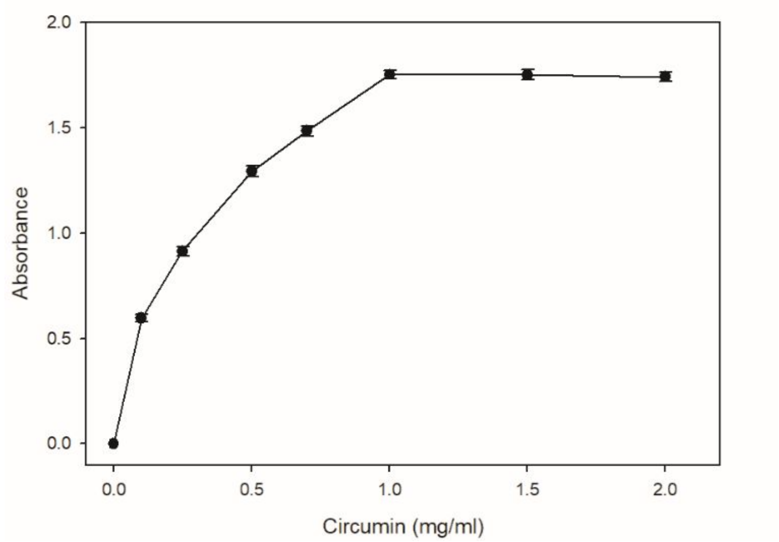

Figure S2. The binding of Zn-Bt using curcumin (1 mg/ml) as mediator to AFs.

(c) Effect of NaOH

To understand whether alkali induce binding of Cur-ZnBt, A_{367} measurement of Cur-ZnBt in presence of different NaOH concentration was performed. As shown in Figure S3, A_{367} value increased steadily till 0.05 M, while it became constant for no change beyond 1 M even after 5 min of reaction. Therefore, we try to avoid the possible saturation effect of NaOH on Cur-ZnBt and fixed the concentration of NaOH as 1 M.

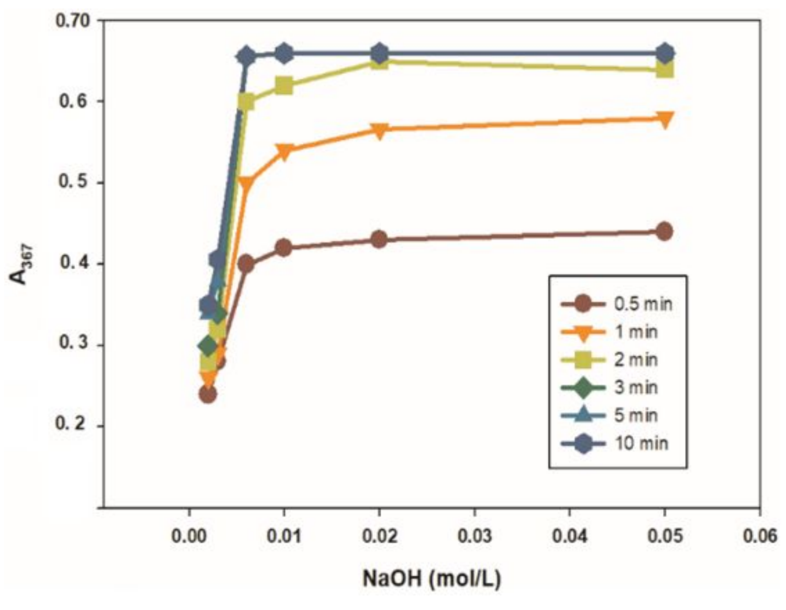

Figure S3. A_{367} measurement of Cur-ZnBt in presence of different NaOH concentration

(d) Incubation time

The effect of incubation time on the colorimetric response was also investigated. Figure S4 shows the binding of Cur-ZnBt with AFs at different pH. The effect of pH at 9.24 was maximum showing maximum value at $A_{367.}$ This effect was found increasing in the initial 2 minutes of the reaction. Thereby, constant value of A_{367} after 2 minutes showed saturation of the bond. Thus initial 2-3 minutes are chosen as the optimized time of incubation and for taking the final measurement at A_{367} as estimated time for the bond formation between AFs and Cur-ZnBt composite.

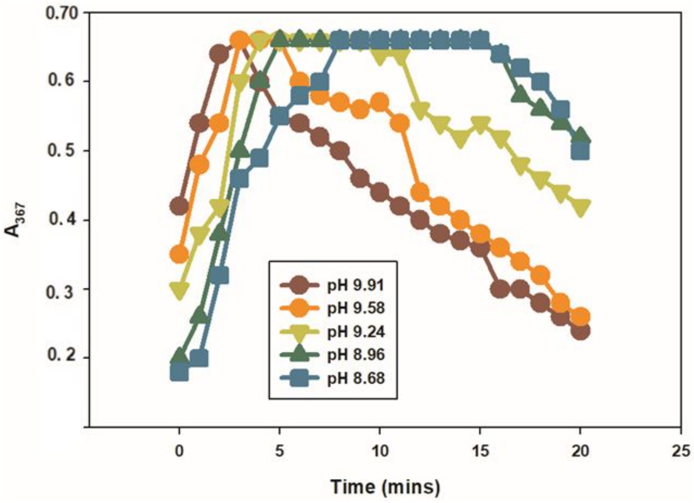

Figure S4. A_{367} value of ZnBt containing curcumin (1mg/ml) versus time at different pH. (The data were recorded at the interval of 1 min).

Table S1. AFs detection in food samples from local and commercial market.

Sample no.	Cur-ZnOBt Detection (ppb)	LC/MS-MS detection (ppb)	Accuracy (%)
1	0	ND	-
2	0	ND	-
3	0.2	ND	-
4	2.74	2.64	96.21
5	4.28	4.02	93.54
6	5.35	5.1	95.09
7	10	8.8	86.4
8	3.19	3.08	96.43
9	20	19.2	95.84

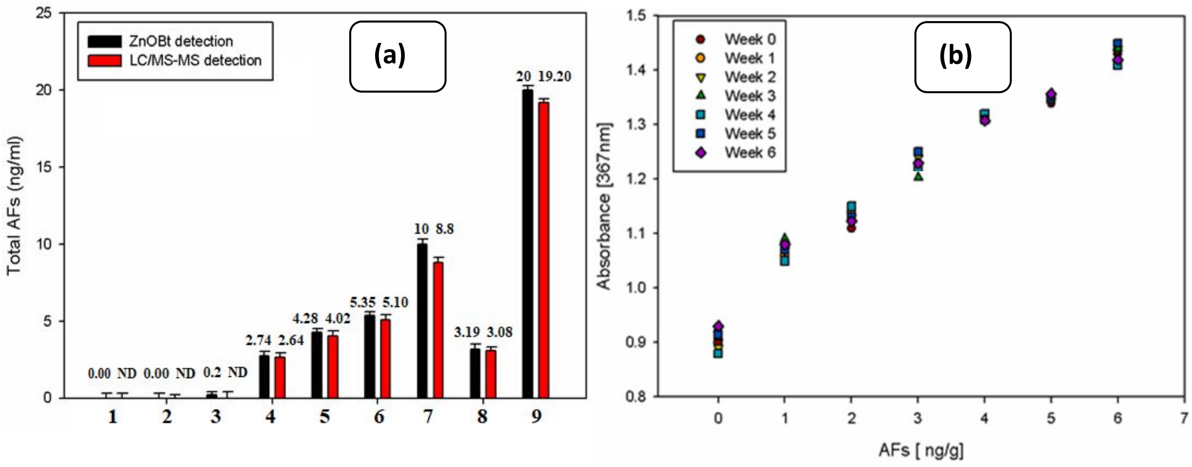

Figure S5 (a) Accuracy calculation of Zn(cur)OBt composite platform in food samples. The real value concentration was verified with standard gold technique LC/MS-MS. (b) Consistency or stability analysis over a period of one week to nine weeks at different AFs concentration from 0.1 to 7 ng/g.

Table S2 CIE L*a*b* coordinates of colorimetric analysis of AFs (AFB_1+AFB_2+AFG_1+AFG_2) in proportion of 1:0.1:0.3:0.03 by the ZnBt -Cur based sensor

Sample	Color	Food sample	L	a*	b*	Comparative interpretation
With respect to Reference (white plate) (n=3)	White		94.84	-0.52± 1.2	4.05± 0.4	It is most light, less redder and more yellower
Control 1- Unspiked corn and almond extract in methanol (ZnOBntNCs -Cur, NaOH, Boric Acid) (n=3)	Red	Corn Almond	20.1 ± 2.2 21.23± 2.4	27.9 ± 1.2 28.1 ± 1.3	17.1 ± 0.4 16.1 ± 0.3	Control 1 It is least light, most red and least yellow than Test
Control 2- Unspiked methanol (ZnOBntNCs -Cur, NaOH, Boric Acid, Methanol)) (n=3)	Red		20.9 ± 1.7	26.7 ± 0.3	18.1 ± 0.32	Control 2 is less light, more red and less yellower than Test

Test- Spiked corn and almond extract (ZnOBntNCs -Cur -AFs, NaOH, Boric Acid) (n=3)	Yellow	Corn<br>Almond	77.9 ± 1.5<br>78.3 ± 1.5	0.5 ± 0.8<br>0.47 ± 0.7	26.5 ± 0.24<br>27.3 ± 0.21	Test is most light, least red and most yellow in colour than control 1 and 2

Analysis of AFs by LC/MS-MS

LC/MS-MS condition:

Detection was performed with A Waters UPLC system (Waters, Milford, MA, USA) equipped with an Agilent JetStream electrospray ionization (ESI) source and a 6470 series Triple Quadrupole LC/MS (Agilent technologies, Germany. Chromatographic separation of AFs was carried out with Kinetex Biphenyl column (50 mm × 3 mm i.d., 2.6 µm particle size) preceded by a Security Guard TM ULTRA Holder pre-column, both supplied by Phenomenex (Phenomenex, Torrance, CA, USA). The flow rate of mobile phase was fixed to 300 µL min^{-1} and the injection volume for both standard and sample was 5.0 µL. The column oven was maintained at 40 C. For MS/MS detection, the ESI interface was used in positive polarity with the following settings: Capillary voltage, source temperature, desolvation gas flow rate, and its temperature were set at 3 kV, 120 ◦C, 600 L h^{-1}, and 350 ◦C, respectively. Collision-induced dissociation was performed using argon as collision gas at a pressure of 3.5×10^{-3} mbar in the collision cell (Figure S5) [3].

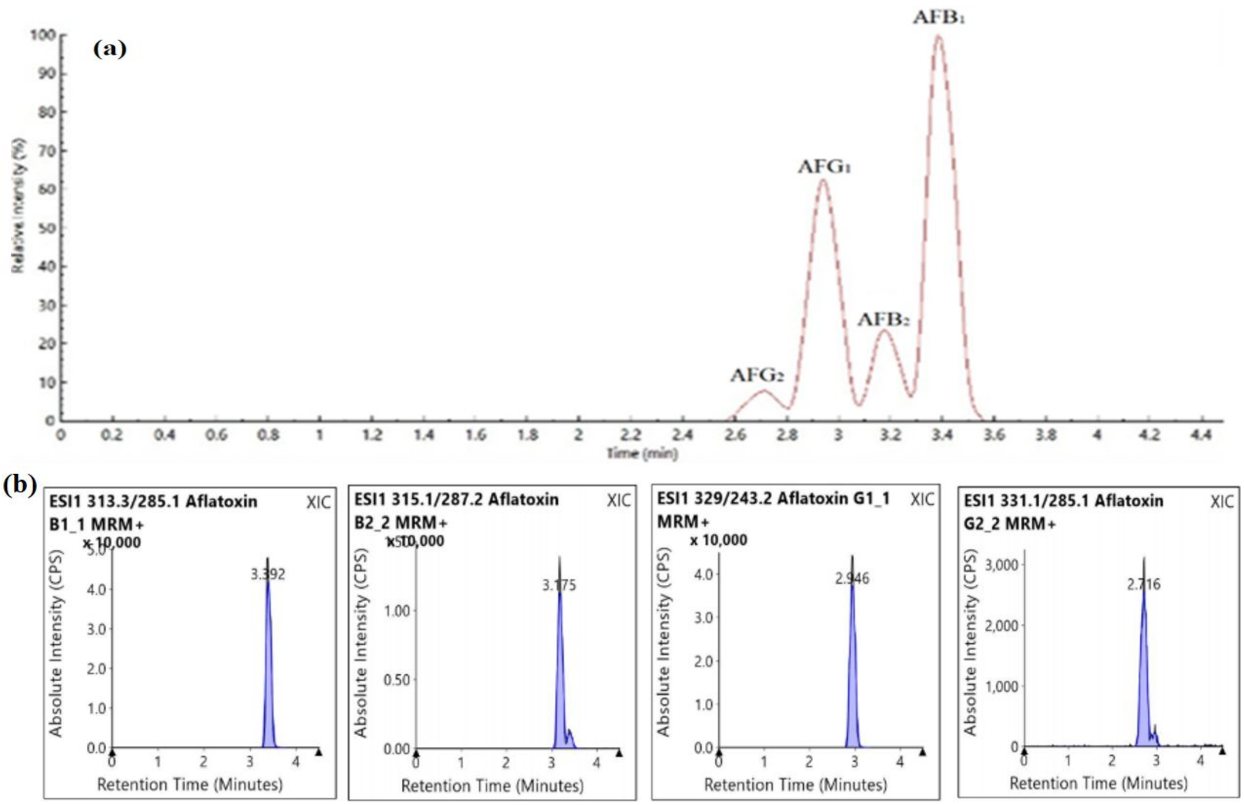

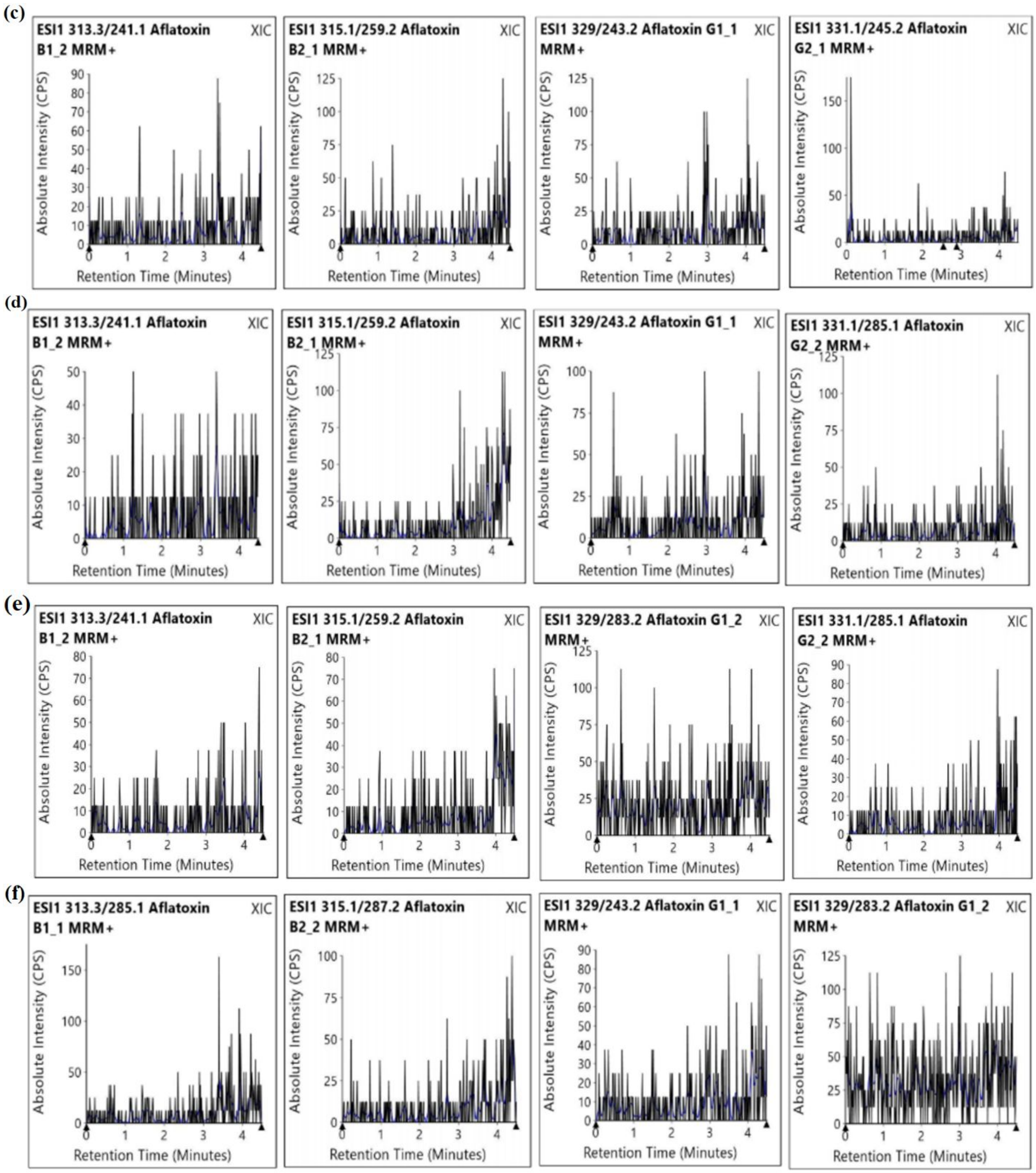

Figure S6. LC/MS-MS chromatograms of (a) AFs chromatogram (b) standard solution (5 ppb) in the ratio of 1:0.1:0.3:0.03, (c) corn sample 1, (d) corn sample 2, (e) almond sample 1, and (f) almond sample 2. Retention times were around 3.392, 3.175, 2.946 and 2.716 min for AFB_1, AFB_2, AFG_1 and AFG_2, respectively. No peak was present in the chromatograms of corn and almond samples, indicating that the selected food samples for addition and recovery experiments were aflatoxin-free.

REFERENCES

1. Nones, J.; Solhaug, A.; Eriksen, G. S.; Macuvele, D. L. P.; Poli, A.; Soares, C.; Nones, J. Bentonite modified with zinc enhances aflatoxin B_1 adsorption and increase survival of fibroblasts (3T3) and epithelial colorectal adenocarcinoma cells (Caco-2). *J. Hazard. Mater.* 2017, *337*, 80–89.
2. Al-Asheh, S.; Banat, F.; Abu-Aitah, L. Adsorption of phenol using different types of activated bentonites. *Sep. Purif. Technol.* 2003, *33*, 1–10.
3. Santis, B. De.; Debegnach, F.; Gregori, E.; Russo, S.; Marchegiani, F.; Moracci, G.; Brera, C. Development of a LC-MS/MS Method for the Multi-Mycotoxin Determination in Composite Cereal-Based Samples. *Toxins* 2017, *9*, 169.
